# A Fortuitous Journey from a Model System to a Human Pathogen

**DOI:** 10.1371/journal.ppat.1005313

**Published:** 2015-12-10

**Authors:** Michael J. Imperiale

**Affiliations:** 1 Department of Microbiology and Immunology and Comprehensive Cancer Center, University of Michigan, Ann Arbor, Michigan

In 1960, Sweet and Hilleman reported the isolation of “The Vacuolating Virus, S.V._40_” from seed stocks of Sabin poliovirus vaccines. In their discussion, they speculated about the possible long terms effects of the introduction of such a virus into the human population, particularly because it was already known that mouse polyomavirus was oncogenic in experimental animals. Indeed, over fifty years later, there remains controversy about a possible role for SV40 in human cancer. Rather than discuss this issue, I wish to focus on the tremendous impact the study of SV40 has made on modern day biology and also my own career.

My first exposure to SV40 (other than potentially through the vaccine; no pun intended) was when I attended my first DNA Tumor Virus meeting in Cold Spring Harbor in 1982. I had just begun studying regulation of adenovirus gene expression as a postdoc. At that time, people were using these small DNA viruses as model systems for understanding various eukaryotic cellular processes. SV40 was particularly important for dissecting eukaryotic DNA replication, based on the finding that its double-stranded circular DNA genome used the host DNA replication machinery. The first completely in vitro replication system using host polymerases was developed using SV40 as a template, and taking advantage of the discovery that the viral large T antigen, or LT, played a crucial role by binding to the origin of replication. In that sense, LT was the first sequence-specific eukaryotic DNA binding protein to be studied. The ability to manipulate the viral genome as a recombinant molecule also led to the isolation of various viral mutants with interesting phenotypes. For example, the study of temperature sensitive mutants led to the finding that LT also regulates transcription of the viral chromosome by the host RNA polymerase II. The first “TATA box” was defined in SV40, as was the first transcriptional enhancer, the infamous 72 bp repeat. As if this were not enough of a contribution to our understanding of eukaryotic gene expression, SV40 was one of two viruses in which mRNA splicing was discovered. Moreover, studies of SV40 transcription demonstrated that pol II proceeds past the site where the poly(A) tail is added, leading to addition of the tail by a post-transcriptional processing event. The AAUAAA 3’ end processing signal was also defined in SV40.

Gene expression is not the only field in which SV40 has left its mark. The first nuclear localization signal was described on LT, leading the way towards detailed understanding of how proteins are imported into the nucleus. The recognition early on that the virus caused tumors when inoculated into newborn hamsters led to its being investigated as a model system for oncogenesis. Early experiments in which investigators immunoprecipitated LT from transformed cells led to the seminal discovery of the now-famous cellular tumor suppressor p53. A variety of other key cell growth control regulators interact with LT.

Little did I know that SV40 was going to end up influencing the rest of my career in a way that I never could have anticipated. One month before I joined the faculty at the University of Michigan in 1984, Bill Brockman, who was studying LT and was instrumental in recruiting me to Ann Arbor, died suddenly. When I arrived on campus, the department chair asked me if I would be willing to step in as Principal Investigator (PI) of Bill’s R01 research grant from the NIH, to allow his students to complete their dissertation research with minimal disruption. I was pretty unsure of what to do. The thought of getting my own independent project on adenovirus up and running was already daunting, and I was faced with the necessity of having to quickly become an expert in a second area. It so happened that the department had organized a symposium in Bill’s honor that was held a couple of months later, and one of the speakers was Dan Nathans, with whom Bill had trained at Johns Hopkins. Dan convinced me that continuing Bill’s work would allow me to contribute to our understanding of the molecular mechanism of cancer. I agreed to become PI of his grant. My excitement about the project grew exponentially, and we later ended up renewing the funding for Bill’s R01 and continuing the work.

In the early 1990’s a graduate student joined the lab and wondered why we weren’t studying a human pathogen. While we both understood the importance of SV40 as a model, I thought that her question deserved an answer, and I asked her to look into the human polyomaviruses. We decided that BKPyV (called BKV at that time) was understudied, and decided to take it on. My initial attempts to fund the work ran into a curious response—namely questions about what we would learn from BKPyV that we didn’t already know from SV40. However, as soon as we began to provide evidence that the two viruses had subtle but important differences, funding came.

I think that one of our key contributions to the field was the realization early on that we should be studying the biology of the virus in its natural host cell. We were fortunate that a colleague at Michigan happened to be developing a cell culture system for primary renal epithelial cells in which the cells would largely maintain their differentiated functions. I learned this completely by accident one day as I was walking down the hall and saw a notice for a seminar he was giving that week. Over the years, we have found differences in viral biology between these cells and other cell lines.

In an era in which the term “translational research” is given a lot of traction, I think that we must not forget that basic research has always yielded, and will continue to yield, critical insights into not only fundamental biology but also potential applications. For example, how long might it have taken to fully understand the importance of p53 in human cancer without the knowledge gained from the SV40 studies? How many years would have gone by before a suitable cellular origin of DNA replication would have been found with which to dissect the DNA synthetic machinery? Models are glamorous for a reason! As is the case with much basic research, I hope that our findings will someday lead to better outcomes for patients with BKPyV disease, but in the meantime I remain convinced that our results follow the tradition of the pioneering studies with SV40—continuing to provide insights into the biology of the virus and its host cell. The phrase “being in the right place at the right time” can often seem trite, but when I think of my serendipitous journey, I feel very fortunate that events largely out of my control, some sad and some not, allowed me to land where I am today.

**Image 1:**
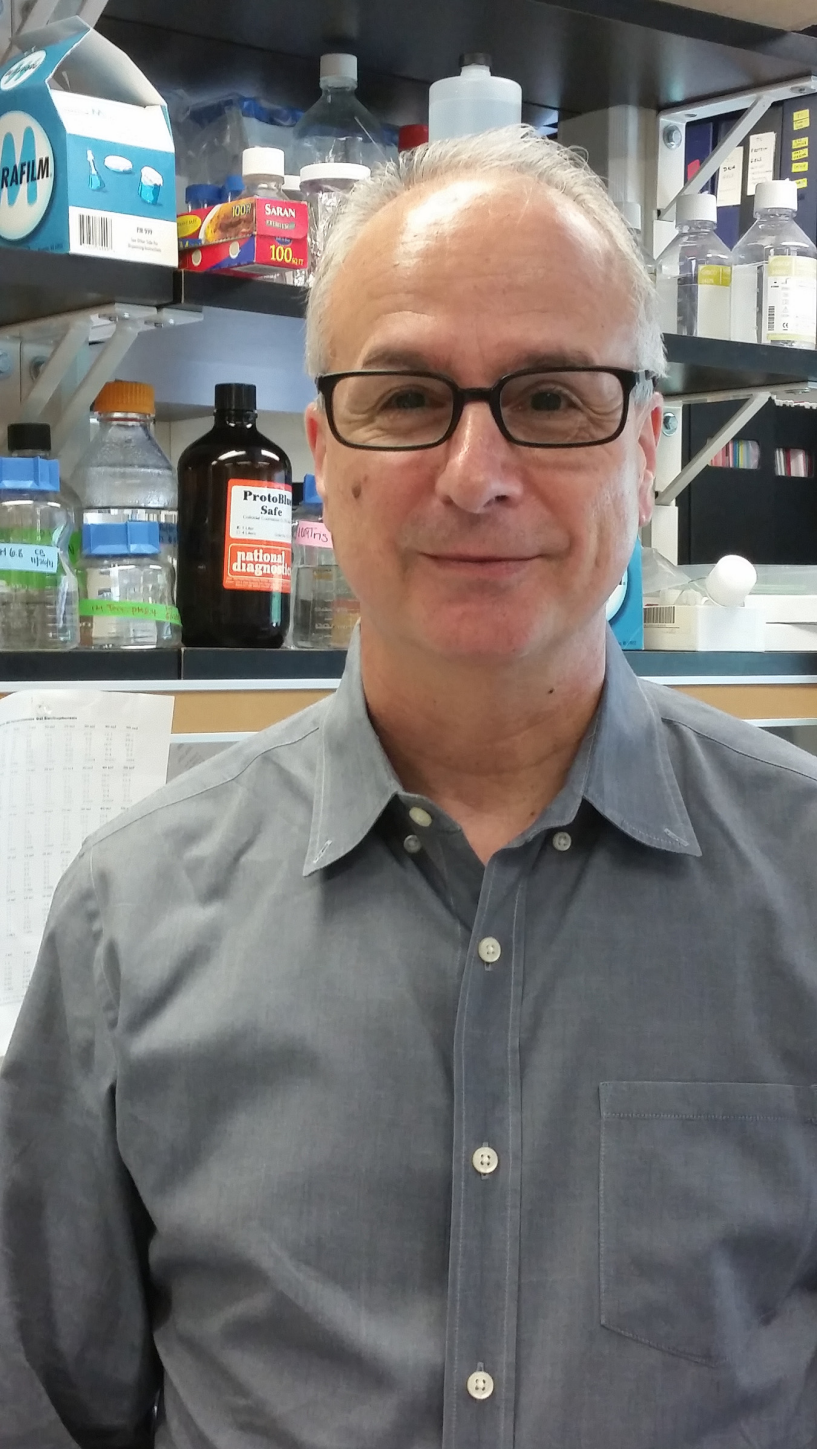
Michael J. Imperiale.

